# Effect of Early Mobilization on Physical Function in Patients after Cardiac Surgery: A Systematic Review and Meta-Analysis

**DOI:** 10.3390/ijerph17197091

**Published:** 2020-09-28

**Authors:** Yuji Kanejima, Takayuki Shimogai, Masahiro Kitamura, Kodai Ishihara, Kazuhiro P. Izawa

**Affiliations:** 1 Department of Public Health, Graduate School of Health Sciences, Kobe University, Kobe 654-0142, Japan; mojaball@yahoo.co.jp (Y.K.); tak813hir@gmail.com (T.S.); pt_masa0808@yahoo.co.jp (M.K.); mhe1601@std.huhs.ac.jp (K.I.); 2 Cardiovascular Stroke Renal Project (CRP), Kobe 654-0142, Japan; 3 Department of Rehabilitation, Kobe City Medical Center General Hospital, Kobe 650-0047, Japan; 4 Department of Physical Therapy, Kokura Rehabilitation College, 2-10 Kuzuharahigashi 2-chome, Kokuraminami-ku, Kitakyushu 800-0206, Japan; 5 Department of Rehabilitation, Sakakibara Heart Institute of Okayama, 5-1 Nakaicho 2-chome, Kita-ku, Okayama 700-0804, Japan

**Keywords:** early mobilization, cardiac surgery, physical function, meta-analysis

## Abstract

The objective effects of early mobilization on physical function in patients after cardiac surgery remain unknown. The purpose of the present study was to clarify the effects of early mobilization on physical function in patients after cardiac surgery through meta-analysis. Four electronic databases were searched on 2 August 2019. We used search keywords related to “early mobilization”, “cardiac surgery”, and “randomized controlled trials”. All randomized controlled trials conducting early mobilization after cardiac surgery were included. We defined early mobilization as the application of physical activity within the first five postoperative days. Citations and data extraction were independently screened in duplicate by two authors. The meta-analysis was conducted using random-effects modeling with EZR software. The primary outcome was the distance walked during the six-minute walking test at hospital discharge. Six randomized controlled trials comprising 391 patients were included following screening of 591 studies. All studies included coronary artery bypass grafting as the cardiac surgery conducted. Early mobilization started on postoperative days 1–2 and was conducting twice daily. Early mobilization showed a trend of being combined with respiratory exercise or psychoeducation. The meta-analysis showed that the distance walked during the 6-min walking test improved by 54 m (95% confidence interval, 31.1–76.9; I^2^ = 52%) at hospital discharge. The present study suggested that early mobilization after cardiac surgery may improve physical function at discharge.

## 1. Introduction

Cardiovascular diseases (CVD) such as coronary artery disease (CAD) are a major cause of mortality worldwide [1]. With rapid growth in the treatment of CVD, cardiac surgery has tended to become more minimally invasive and has reduced mortality from CVD [2]. However, the risk factors for postoperative complications from cardiac surgery still remain high [3]. Postoperative rest-centered management of pain, dyspnea, sleep problems, and depression can cause postoperative complications related to bed rest [4]. Bed rest contributes to decreased cardiac output, secondary complications such as deep venous thrombosis, pneumonia, and pressure sores, loss of muscle mass and strength, and a decline in aerobic capacity within the first few postoperative days [5,6]. Therefore, effective countermeasures to bed rest after cardiac surgery must be developed. Early mobilization has attracted attention as one of the countermeasures to bed rest. 

Early mobilization is defined as the application of physical activity within the first two to five days of critical illness or injury [7,8]. In critically ill patients, early mobilization has significant effects on the length of intensive care unit (ICU) and hospital stays, ICU-acquired weakness, the Barthel index as an index of the activity of daily living, and the incidence of complications [9,10]. Although early mobilization was safe and well tolerated, 12 patients were reported to initially feel “too sick” or were thought to be “very clinically unstable” for mobilization [11]. The evidence level for early mobilization is still poor, indicating a gap in perception between medical staff and patients. Therefore, further evidence needs to be acquired regarding the safety and effectiveness of early mobilization. 

A decline of physical function (gait speed) with bed rest was seen in 17% of patients after surgery [12]. Furthermore, physical function (handgrip strength, knee extensor muscle strength, 6-min walking test (6MWT), and peak oxygen intake) of CVD patients is related to all-cause mortality, hospital admission, and major adverse cardiovascular events [13,14]. The decline of physical function can be predicted by several clinically modifiable factors [12]. These modifiable factors related to physical function can be assessed with the 6MWT, which is related to the type of cardiac surgery, the functional independence measure (FIM), and body mass index [15]. Use of a cut-off point of <300 m in the 6MWT is a simple and useful prognostic marker of mortality and readmission [16,17]. Therefore, interventions to counter bed rest such as early mobilization may be necessary to prevent the decline of physical function after cardiac surgery. 

Ramos Dos Santos et al. found that three quarters of the studies related to cardiac surgery that they analyzed reported early mobilization to have significant effects on improving physical function such as that assessed in the 6MWT [18]. However, a meta-analysis was not conducted due to heterogeneity between the early mobilization protocols used in the included studies. Few meta-analyses have been published on the effects of early mobilization in patients after cardiac surgery. We hypothesized that early mobilization of patients after cardiac surgery would improve physical function (as assessed by the distance covered in the 6MWT) at discharge. The purpose of the present study was to clarify the effects of early mobilization on physical function in patients after cardiac surgery through a systematic review and meta-analysis of previous related studies.

## 2. Materials and Methods 

### 2.1. Eligibility Criteria

This systematic review and meta-analysis was conducted according to the PRISMA statement [19]. We included randomized controlled trials (RCTs) studying early mobilization in patients after cardiac surgery. We imposed no restrictions on language, publication date, and publication status. Patients over 18 years of age undergoing open cardiac surgery (i.e., coronary artery bypass grafting [CABG], aortic valve replacement) were targeted. The intervention was early mobilization defined as the application of physical activity (i.e., passive range of motion and ambulation) within the first 5 postoperative days [7,8]. We imposed no restrictions on frequency, intensity, or type and time of intervention. The primary outcome was the distance achieved during the 6MWT at hospital discharge.

### 2.2. Search Strategy

**Figure 1:**
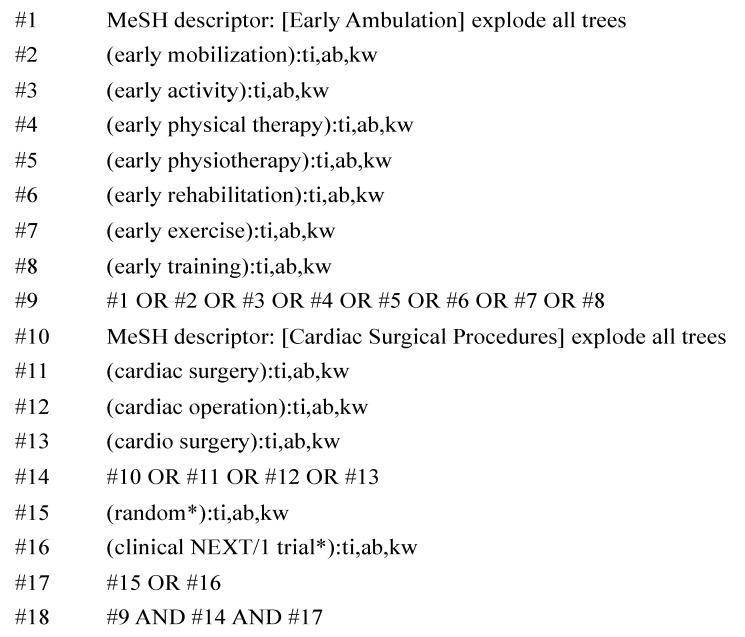
Search strategy in the present study. Legends: MeSH, medical subject headings; ti, title; ab, abstract; and kw, keyword.

Studies were identified by searching four electronic databases (PubMed of the U.S. National Library of Medicine, Scopus, Cochrane Controlled Register of Trials, and Web of Science). We searched for keywords related to “early mobilization”, “cardiac surgery”, and “randomized controlled trials” (Figure 1). The last search was conducted on 2 August 2019. Four studies were added by manual research after referring to the Ramos Dos Santos et al. study [18]. Several studies for which the full text was unavailable were excluded.

### 2.3. Study Selection

Eligibility assessment was performed independently in a standardized unblinded manner by two independent reviewers (Y.K. and T.S.). First, we screened the titles and abstracts of each study. After the results of this screening were integrated, we screened the full text of the manuscripts. Disagreements regarding inclusion of a study were reconciled via consensus.

### 2.4. Data Extraction

We used datasheets based on the Cochrane Consumers and Communication Review Group’s data extraction template. Two reviewers independently extracted the data listed below from the included studies. After that, each result was integrated, and disagreements were resolved by discussion between the two independent reviewers (Y.K. and T.S.). If agreement could not be reached, we requested intermediation by a third party. We contacted seven authors directly to collect additional data. After that, three full texts and the details of the results of two studies were collected and included in the present analysis. The following information was extracted from the included studies: patient characteristics (including age, sex, country, diagnosis, comorbidities, type of surgery, and duration of ICU stay), interventions (including frequency, intensity, time, type, start date, and interventions in controlled groups), and outcomes (including distance walked and postoperative day the 6MWT was conducted).

### 2.5. Quality Assessment

To assess the validity of the included studies, two reviewers independently used the “Cochrane risk of bias tool”, which consists of seven items that were evaluated as “low risk”, “high risk”, or “unclear risk” and are summarized in Table 1 [20]. The present study complied with the principle of the Declaration of Helsinki regarding investigation in humans.

### 2.6. Statistical Analysis 

The distance walked during the 6MWT at discharge was the primary outcome of the intervention effect. In the meta-analysis, we calculated the mean distance walked during the 6MWT in each group at discharge using the weighted average “random-effect model” included in the EZR analysis software [21]. We judged inconsistency in the results between the studies as indicating heterogeneity if the *p*-value was < 0.05 in the Cochrane Q test or >50% in the I^2^ test as a measure of inconsistency. No other additional analyses were conducted.

## 3. Results

### 3.1. Overview of Included Studies

**Figure 2:**
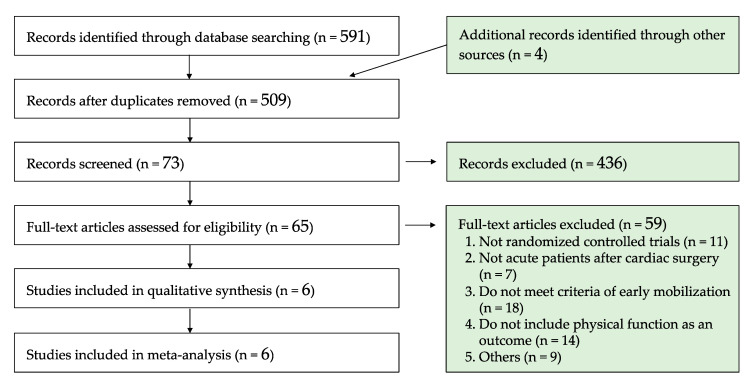
Flow diagram of study selection.

We searched 591 studies through four database searches along with four additional studies obtained from the Ramos Dos Santos et al. study [18]. After duplicates were removed, we screened 509 titles and abstracts and initially excluded 436 studies. After screening the remaining 65 full-text articles, 6 RCTs were selected for inclusion in this systematic review and meta-analysis (Figure 2) [22,23,24,25,26,27]. Among the six included studies, the oldest study was published in 2008. Two studies were conducted in Denmark, and the other four studies were conducted in Brazil. There were no duplicate patients in the included studies. Mean age of the included patients ranged from 58.5 to 65.1 years, and the ratio of females ranged from 13.5 to 45.0%. Mean left ventricle ejection fraction of the patients reported in two studies ranged from 48.0 to 63.0% [25,26], and almost all patients were classified as New York Heart Association class I–III. The patients were diagnosed as having CAD or ischemic heart disease, and all patients underwent nonemergent isolated CABG. In addition, if patients underwent additional surgery after CABG, they were excluded from the protocol of the included studies. Patients with surgical reintervention, mediastinitis, stroke or resuscitation from cardiac arrest after surgery, and those who died during/after surgery were excluded in the included studies. Patients were hospitalized for 5–7 days after CABG and underwent a 6MWT conducted at discharge. The 6MWT was conducted in a 30-m corridor by physiotherapists or nurses.

### 3.2. Trend of Interventions

Early mobilization started on postoperative days 1–2 and was conducted twice daily by physical therapists or nurses. The exercise protocol was performed via a progressive approach, and it consisted of such activities as active upper and lower limb exercise training, ambulation, cycle ergometer, and ascent/descent of stairs. Ambulation was started by postoperative day 1 or 2 in almost all studies. Interventions in four studies were continued until discharge, although in two studies, they were continued until 4 months after surgery. Respiratory physiotherapy (e.g., deep breathing exercises, incentive spirometry, and inspiratory muscle training) and psychoeducation were implemented as additional interventions with early mobilization. The aim of psychoeducation was to improve disease coping skills (e.g., sleep disorders and physical and emotional stress) by applying a patient-centered approach. These interventions in two of the studies were conducted in the same institution. In addition, the psychoeducation was conducted four times per patient by trained nurses and was based on the Human Becoming Practice Methodologies [28]. Herdy et al. reported that early mobilization resulted in a reduction in the incidence of postoperative pneumonia, atrial fibrillation, plural effusions, and atelectasis [27]. Two episodes of ventricular tachycardia occurring after the 6MWT were reported [22], but otherwise, there were no reports of adverse events in any of the early mobilizations after cardiac surgery.

### 3.3. Risk of Bias

**Figure 3:**
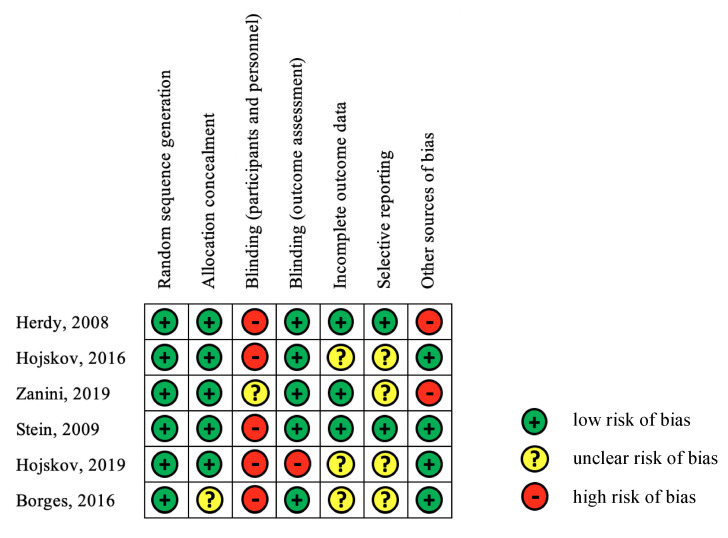
Summary of the risk of bias in the analyzed studied.

Figure 3 summarizes the results of the assessment of the risk of bias in the six studies. The tool used to assess risk of bias considered random sequence generation and blinded outcome assessment, and as a result, the assessment indicated a high ratio for the low risk of bias. However, a high percentage of the blinded participants and personnel showed high risk of bias. The included studies tended to show an unclear risk of bias in terms of incomplete outcome data and other sources of bias.

### 3.4. Meta-Analysis

**Figure 4:**
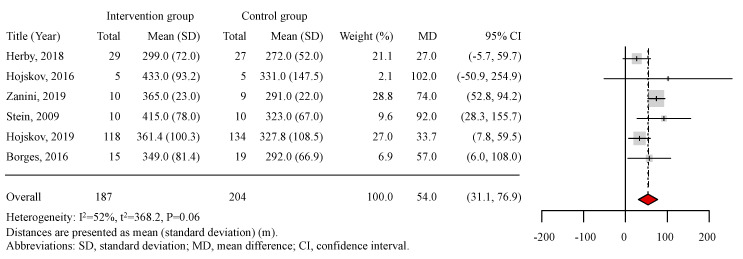
Meta-analysis. Distances are presented as mean (standard deviation) (m). Legends: SD, standard deviation; MD, mean difference; and CI, confidence interval.

In the meta-analysis of all included studies, the distance walked during the 6MWT at discharge was set as the objective variable. In total, 391 patients were included in the analyzed studies (187 patients in the intervention group and 204 patients in the control group, in whom usual care was conducted). The results of the meta-analysis are shown in Figure 4. Four of the six included studies showed significantly positive efficacy for early mobilization. The mean distance walked during the 6MWT in the intervention group ranged from 299.0 to 433.0 m, whereas that in the control group ranged from 272.0 to 331.0 m. Patients in the intervention group walked longer and farther during the 6MWT than those in the control group in all included studies. According to the results of the mean differences, early mobilization resulted in an increase of 54.0 m walked (95% confidence interval: 31.1–76.9 m) during the 6MWT at discharge. The result of the I^2^ test was 52%, and the P-value for heterogeneity was 0.06, and although these results indicated moderate heterogeneity in the six studies, early mobilization significantly increased the distance walked during the 6MWT at hospital discharge.

## 4. Discussion

In the present meta-analysis, early mobilization was shown to clinically significantly improve physical function at discharge. To our best knowledge, this is the first meta-analysis to examine the effectiveness of early mobilization on physical function in patients after cardiac surgery.

### 4.1. Comparison with Previous Studies

In a previous systematic review, early mobilization was shown to have a positive effect on improving physical function [18]. The outcomes of the present meta-analysis are in line with this previous study. Importantly, the present meta-analysis revealed additional data showing that early mobilization resulted in patients increasing the distance walked during the 6MWT by 54 m after cardiac surgery. Respiratory exercise in all six studies and psychoeducation in two studies were conducted along with early mobilization, although one previous study reported that respiratory exercise alone does not have a significant effect on physical function after cardiac surgery [29]. As such, these findings suggest that early mobilization is more effective than other interventions in improving physical function after cardiac surgery. In other areas such as the ICU, early mobilization has also contributed to the improvement of physical function [10], but safety considerations related to respiratory, cardiovascular, neurological, and other issues must be considered before conducting early mobilization [30]. In detail, respiratory considerations include intubation status, adjunctive therapies, and ventilatory parameters; cardiovascular considerations include the presence of devices, cardiac arrhythmias, and blood pressure; neurological considerations include level of consciousness, delirium, and intracranial pressure; and other considerations include intravenous lines and surgical or medical conditions [30]. In this context, interventions that focus on early mobilization along with safety considerations are indicated to improve physical function after cardiac surgery.

### 4.2. Possible Explanation and Implications

According to previous studies, the minimum important clinical difference in the 6MWT is an increase in distance walked of 25 m for patients with CAD and 14.0–30.5 m for patients with other diseases [31,32]. The present meta-analysis showed that early mobilization resulted in an increase in the mean difference of 54 m in the 6MWT compared to the control groups. Therefore, early mobilization may lead to a clinically important difference at discharge. Previous studies have shown that the longer the distance walked in the 6MWT, the shorter is the time of hospitalization [33]. In addition, a distance of <300 m in the 6MWT suggests severe exercise intolerance and is a prognostic marker of mortality and readmission [16,17]. The patients undergoing early mobilization in the present meta-analysis walked from 299 to 433 m, indicating that almost all patients exceeded 300 m. This suggests that their improved physical function may result in lower mortality and a lower rate of readmission following discharge [13,14].

Bed rest induces changes in skeletal muscle atrophy and inflammatory markers [34]. Cardiac surgery is an invasive treatment that increases markers of inflammation that cause muscle catabolism in the postoperative phase. However, one previous study showed that muscle activity serves as an anti-inflammatory action [35]. Therefore, promoting muscle activity through early mobilization may prevent muscle catabolism and physical dysfunction. In addition, bed rest also results in a decrease of peak oxygen intake as a function of aerobic capacity that is associated with decreased stroke volume and cardiac output [36,37]. Taken together, early mobilization, which counters the effects of bed rest, may prevent a decrease in aerobic capacity, and patients undergoing early mobilization might walk a longer distance during the 6MWT as a result of the prevention of dysfunction due to bed rest.

The present analysis showed that not only early mobilization but also respiratory exercise and psychoeducation were conducted as interventions after cardiac surgery. Respiratory dysfunction and depression are reported likely to occur after cardiac surgery [38,39]. In the studies in the present meta-analysis, respiratory exercise included deep breathing exercises and incentive spirometry, and psychoeducation was conducted to improve disease coping skills based on the Human Becoming Practice Methodologies [22,23,25,26,27]. Comprehensive cardiac rehabilitation programs may improve physical and psychological problems [40], so our findings suggest that the combination of early mobilization and these interventions may improve physical function even more.

None of the analyzed studies reported adverse events during early mobilization. Serious adverse events are uncommon during early mobilization when safety considerations are addressed prior to mobilization [10,30]. Taken together, this result was in line with previous studies suggesting that patient anxiety regarding early mobilization after cardiac surgery may be unfounded.

### 4.3. Limitations

There are several limitations in the present meta-analysis. First, only six studies were included, and these studies were conducted only in Brazil and Denmark. In addition, two of the six studies were from 10 years ago. As the technology and context of cardiac surgery have continued to advance in the last decade, such as in the reduction of its invasiveness, the background of these older studies is likely to be different. Although the present analysis could not clarify this, the combination of current improvements in cardiac surgery and earlier mobilization may have resulted in a better postoperative prognosis compared to the results reported here. Moreover, this meta-analysis included only 391 patients after cardiac surgery, and the female ratio was 13–45%. In the present meta-analysis, about 60% of the patients were from the studies of Hojskov et al., and thus, this could influence the results of the meta-analysis, which included only six studies. Based on the above results, this meta-analysis shows heterogeneity, and the possibility of overestimation may be present. 

Second, only patients diagnosed as having CAD or ischemic heart disease and underwent CABG only were included. In addition, the present study could not distinguish whether on-pump or off-pump CABG was performed. If off-pump CABG is conducted, patients benefit from early discharge and recover their physical function better at hospital discharge. On the other hand, to the best of our knowledge, there are few RCTs examining the effects of early mobilization after other types of cardiac surgery, such as aortic valve replacement and mitral valve plasty, on physical function. The type of cardiac surgery is a determinant of distance walked during the 6MWT [15], so further studies involving patients after cardiac surgery other than CABG should be analyzed. 

Third, the effect of early mobilization alone was not considered because respiratory exercise and psychoeducation were also conducted as interventions along with early mobilization in the included studies. On the other hand, early mobilization suggests a comprehensive intervention that includes respiratory exercise and psychoeducation. These interventions will be performed in almost 100% of postcardiac surgery patients in the clinical setting. However, the novelty of the present meta-analysis was to clarify the effect of these interventions on improving physical function at hospital discharge. In addition, in almost all included studies, the difficulty of blinding the participants and personnel to early mobilization itself indicated a high risk of bias. 

Fourth, only the 6MWT was included in the present assessment of physical function. Other assessments of physical function (e.g., handgrip strength, knee extensor muscle strength, and peak oxygen intake) were included in the analyzed studies, and thus, these indexes should also be studied. 

Finally, the primary outcome was assessed only at discharge from hospital, and the effects of early mobilization on long-term factors of prognosis such as mortality and hospital readmission were not considered. In fact, one of the included studies showing significant differences in the 6MWT between the intervention and control groups reported that the significant difference in the 6MWT was lost at four weeks after discharge [22]. As a next step, further studies should consider the effects of early mobilization on long-term prognosis and other physical functions. The positive effects of early mobilization after cardiac surgery are well known in the clinical setting. However, to further improve the level of evidence, the effects of early mobilization should be revealed based on objective indices. In addition, we would like to perform a meta-analysis to examine whether different types of cardiac surgery have a different effect on early mobilization compared to those of CABG.

## 5. Conclusions

By integrating the data of RCTs of early mobilization for physical function in patients after cardiac surgery, the findings of the present meta-analysis underscore the fact that early mobilization after cardiac surgery may improve physical function (distance walked during the 6MWT) at hospital discharge. Five of the six included studies showed a significantly positive effect of early mobilization, and no adverse events occurred during early mobilization after cardiac surgery. Early mobilization after cardiac surgery also tended to be combined with respiratory exercise and psychoeducation. Further study is required to examine the effectiveness of early mobilization with increased numbers of studies and patients and for other types of cardiac surgery.

## Figures and Tables

**Table 1:** Summary of included studies.

Study/Country	Sample Size	Mean Age, Years	% Female	Surgery/Diagnosis	Intervention	Start Day/Frequency	Duration
Herdy, 2008 Brazil [22]	56	59.5	17/56 (30%)	CABG/CAD	Aerobic training (ambulation: 2-4 METs) Ascent/descent of stairs Respiratory exercise (spirometer training, intermittent positive pressure breathing)	POD 1/NR	Started from at least preoperative day 5 and continued to discharge.
Hojskov, 2016 Denmark [23]	60	64.8	13/60 (22%)	CABG/CAD	Aerobic training (ambulation, stationary bicycle) Muscle exercise (sit to stand, heel lift) Respiratory exercise Psychoeducation	POD 1/twice daily	Started at admission and continued to 4 weeks after CABG
Zanini, 2019 Brazil [24]	40	58.5	11/40 (28%)	CABG/CAD	Aerobic training (ambulation) Limb exercise Respiratory exercise (inspiratory muscle training)	POD 2/twice daily	Started preoperatively and continued to discharge
Stein, 2009 Brazil [25]	20	63.5	9/20 (45%)	CABG/CAD	Aerobic training (ambulation) Limb exercise Ascent/descent of stairs Respiratory exercise	POD 1/NR	Started preoperatively and continued to discharge
Hojskov, 2019 Denmark [26]	326	65.1	42/326 (13%)	CABG/Ischemic heart disease	Aerobic training (ambulation, stationary bicycle) Muscle exercise (sit to stand, heel lift) Respiratory exercise Psychoeducation	POD 1/NR	Started at admission and continued to 4 weeks after CABG
Borges, 2016 Brazil [27]	34	62.7	12/34 (35%)	CABG/CAD	Aerobic exercise (ambulation, stationary bicycle) Limb exercise Respiratory exercise	POD 1/twice daily	Started preoperatively and continued to discharge

Legends: CABG, coronary artery bypass grafting; CAD, coronary artery diseases; NR, not reported; and POD, postoperative day.
